# Is online self‐regulatory training effective in weight control? A pilot experiment on adolescence obesity during coronavirus‐19 lockdown

**DOI:** 10.1002/brb3.2772

**Published:** 2022-10-09

**Authors:** Asiyeh Rezaei Niyasar, Alireza Moradi, Narges Radman, Meysam Sadeghi, Maryam Mahmoudi

**Affiliations:** ^1^ Cognitive Psychology Department Institute for Cognitive Science Studies Tehran Iran; ^2^ Department of Clinical Psychology Kharazmi University Tehran Iran; ^3^ School of Nutrition and Dietetics Tehran University of Medical Sciences Tehran Iran; ^4^ School of Cognitive Sciences Institute for Research in fundamental Sciences Tehran Iran; ^5^ Department of Psychiatry Roozbeh Hospital, Tehran University of Medical Sciences Tehran Iran

**Keywords:** adolescence, executive functions, obesity, self‐regulation

## Abstract

**Objective:**

Studies have shown that obesity is associated with decreased executive function. Impaired executive functions lead to poor self‐regulation, which in turn may result in persistence of unhealthy behaviors, including eating behaviors, throughout life. Increasing self‐regulation in childhood and adolescence has positive effects on creating healthy behaviors such as reducing unnecessary eating and changing unhealthy eating habits. The main purpose of this study is to evaluate an intervention package based on cognitive self‐regulation training in changing eating behaviors and reducing obesity in children and adolescents.

**Methods:**

Fifty‐six students with obesity aged 12–16 years participated in the study in three groups (cognitive self‐regulation training [CSRT], diet, and control). The CSRT group received twenty 30‐min online training sessions with a diet over 10 weeks. The diet group received only a diet with no other intervention, and the control group did not receive any intervention.

**Results:**

The results of our 2 × 3 repeated‐measures ANOVA showed that the CSRT group had a mean BMI decrease of 2.21 (kg/m^2^) after ten weeks, and 3.24 (kg/m^2^) at the follow‐up time. The diet group had a BMI decrease of 0.49 (kg/m^2^) at the ten weeks. In addition, the results showed that the CSRT had a significant reduction in eating behaviors such as external eating and emotional eating. However, the other two groups showed no changes in eating behaviors.

**Conclusions:**

Our results show that online cognitive self‐regulation training has been effective in weight loss and eating behaviors. This study shows promising evidence for the efficacy of the online CSRT‐training as a weight stabilization intervention in children with obesity.

## Graphical Abstract

This study aimed to design an intervention model promoting self‐regulation of cognitive functions in order to modulate eating behaviors and weight control. We recruited a total of 56 adolescents with obesity and randomly assigned them to three groups: Cognitive Self‐Regulation training (CSRT), diet and control groups. Our results show that online cognitive self‐regulation training has been effective in weight loss and changing eating behaviors.

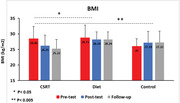

## 1 INTRODUCTION

Obesity and overweight are defined as the accumulation of excess fat in the body, which disrupts a person's health (Favieri et al., 2019). At least 10% of children worldwide are overweight or obese (Wang & Lobstein, 2006).

Overweight and obesity can affect the quality of children and adolescents’ lives (Rafaela et al., 2020; Wille et al., 2008). Overweight in childhood significantly predicts obesity in adulthood; in addition, childhood obesity is associated with increased mortality and various diseases such as type 2 diabetes, cardiovascular disease, as well as orthopedic and respiratory diseases (Flegal et al., 2013; Koletzko et al., 2020; Ward et al., 2017).

Some important factors which lead to excessive weight gain include “emotional eating” (i.e., eating to deal with negative emotions) and “external eating” (i.e., the eating behavior in response to external symptoms such as the image or smell of food). Besides, restrained eating refers to behaviors to limit eating (Nagl et al., 2016; Van Strien et al., 2012). According to Nagl et al. (2016), restraint dieters suppress their hunger using cognitive control. When cognitive control is disrupted, restrained eaters tend to eat more than nondieters. Lifestyle modifications and eating healthy foods along with getting enough exercise can significantly reduce obesity (Hill et al., 2003). However, while millions of people go on a diet, most of them eventually gain weight (De Ridder et al., 2014). One of the key elements of being able to resist the temptations of delicious foods and achieve the long‐term weight loss goal is self‐regulation. Self‐regulation is defined as a cognitive capacity with the help of which a person can resist temptations and impulsivity, and control his thoughts, actions, and even emotions to achieve a specific goal (Blair, Clancy & Diamond, 2008, Diamond, 2013). The ability to control the motivation to eat tasty, high‐calorie food is essential for regulating eating behaviors. Previous studies have shown a link between response inhibition impairment and unhealthy eating habits such as overeating in response to external food cues or negative emotional states (Guerrieri et al., 2007; Jasinska et al., 2012; Nederkoorn et al., 2010). Obesity is associated with problems of self‐regulation (Miller et al., 2017). Besides impaired self‐regulatory capacity, studies show an inverse relationship between increased body mass index (BMI) and executive dysfunction in adults (Gunstad et al., 2008; Nilsson & Nilsson, 2009). Executive functions consist of several higher‐order cognitive processes that allow individuals to perform and manage purposeful actions (Hofmann et al. 2012; Houben et al. 2016; Miyake et al., 2000). The main views in this area suggest inhibitory systems (also known as self‐control), working memory, and cognitive flexibility (which includes task switching/shifting) as the core components of executive functions (Diamond, 2013). These three components begin to develop during childhood and are nearly completed by early adolescence and adolescence (Best et al. 2009).

Working memory (i.e., holding and mentally mediating information) also importantly affects eating behavior. It helps the person integrate knowledge and behavioral skills in achieving a long‐term goal to eat healthily. It also helps them maintaining the capacity to resist short‐term food desires (Hofmann et al., 2008; Hofmann et al. 2007).

Cognitive flexibility refers to the rapid changes in thought and behavior in response to changes in the environment. Concerning eating behavior, cognitive flexibility means the ease of using coping strategies when faced with a new and unexpected temptation to eat (Diamond, 2013; Hayes et al., 2018). Studies have shown that in addition to the above‐mentioned top‐down executive functions processes, bottom‐up processes such as attention bias and delay discounting that interact with executive functions, also affect self‐regulatory skills (Davidson et al., 2015; Hayes et al., 2018).

Inhibitory control is the ability to suppress a prepotent response and save a desired reaction when there is interference from competing events. Regarding weight control, inhibitory control helps avoiding tempting foods, such as saying no to a piece of cake in a party or choosing healthier snacks (Hayes et al., 2018).

Thus, based on the findings emerged from previous studies in the field, the effect of executive function performances on eating behavior, mainly through self‐regulation, is considered crucial in the treatment of weight loss and obesity. The primary aim of this study was investigating the effect of cognitive training focused on eating behavior (cognitive self‐regulation training [CSRT]) in children and adolescents with obesity. As our secondary aim, we planned to investigate the effect of CSRT and diet on weight loss in this population. We hypothesize that children and adolescents receiving CSRT will (1) undergo changes in eating behaviors and (2) a decrease in BMI. We also expect that these changes in eating behaviors and BMI are more prominent in CSRT than in other groups.

## 2 METHODS

### 2.1 Study population

The statistical population in the present experimental study includes all students with overweight and obesity aged 12–16 in the middle school of Tehran in the academic year 2019–2020.

Ninety adolescents volunteered to participate in this study through advertising in high schools in Tehran and on social media. Inclusion criteria consisted of (1) adolescents aged between 12 and 16 years old; (2) obesity as defined with the body mass index (BMI) ≥85th percentile for the age and gender; (3) no physical illnesses such as heart disease, malignancies, diabetes mellitus, other hormonal diseases, and liver failure as were evaluated using a checklist; (4) no psychiatric disorders such as developmental or learning disabilities, attention deficit hyperactivity disorder (ADHD), depression, anxiety, obsessive‐compulsive disorder (OCD), and any history of psychiatric medication (these conditions were assessed through a clinical interview by a clinical psychologist); (5) not receiving any other psychotherapy services regarding weight control; (6) a lower than a cut‐off score of anxiety, depression, and stress of the Depression Anxiety Stress Scale (DASS) (i.e., cut‐off scores of 14, 20, and 25, respectively) (Fathi Ashtiani, & Dastani, 2009); and (7) a score of 20 or less on the Eating Attitude Test (EAT‐26). Seventy‐eight adolescents filled in the inclusion criteria; among them 56 participants completed all the parts of the study.

### 2.2 Study procedure

At first, we conducted a pilot implementation of a CSRT. After conducting the initial assessments (pretest questionnaires and measuring weight, height, and body size) and obtaining the consent of parents and students, the pilot implementation started for seven people. Two participants stopped the intervention in the middle of the work, and finally, five participants were treated entirely. At the pilot stage, the intervention sessions were held in 1‐h sessions per week in individual appointments with the educator. In each session, in addition to the topics determined, the subjects’ weight was measured. The topics and information of each session were written for each person in his/her handouts. After 10 sessions, the initial evaluations were repeated. Thus, the feasibility of the designed package was evaluated in the pilot phase (Rezaei Niyasar, 2021).

**FIGURE 1:**
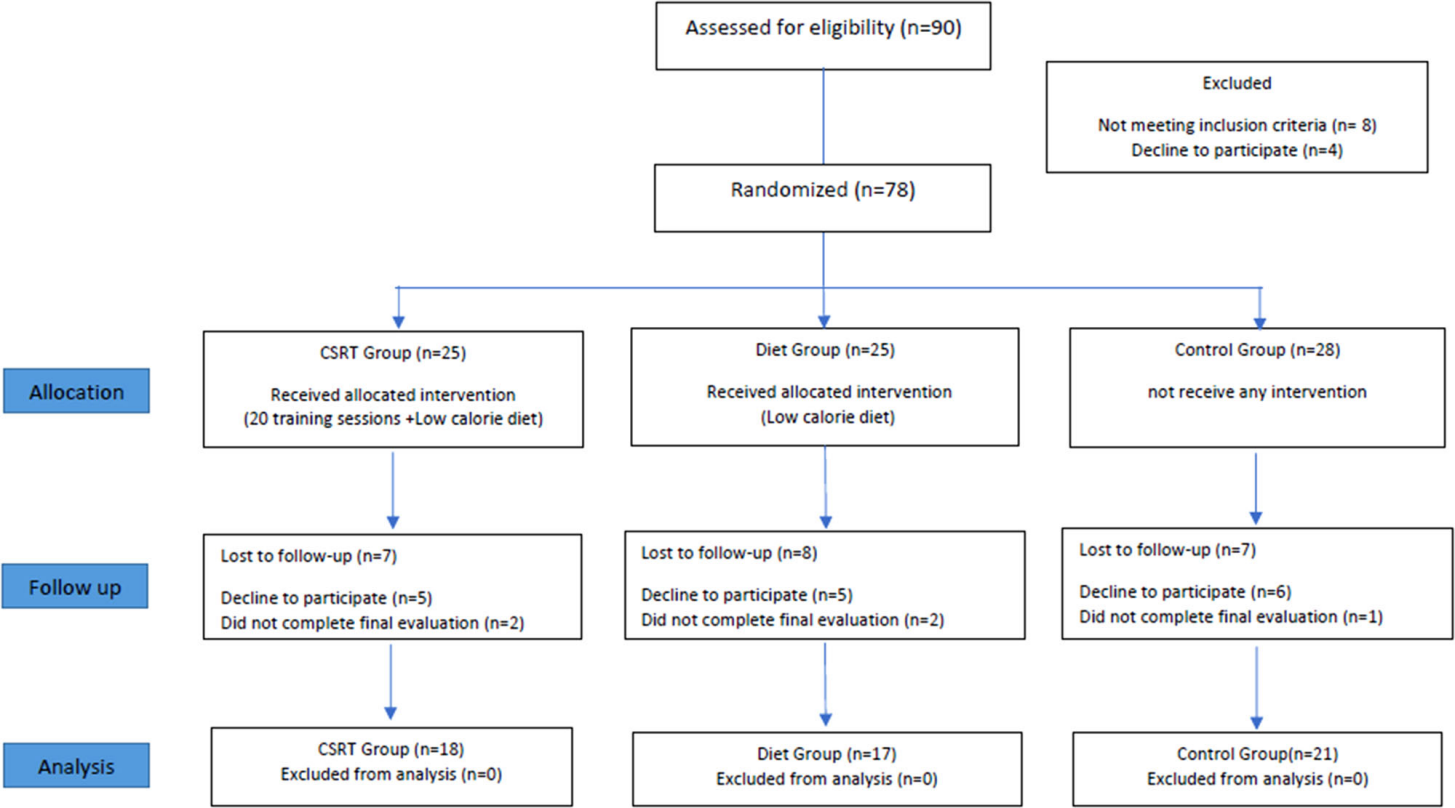
Recruitment flow diagram showing numbers of participants included in each intervention group at each stage of the study

In the main phase of the study, initially 78 participants were randomly assigned into the following three groups: cognitive self‐regulation training (CSRT), diet, and control groups. Participants in the CSRT group received both a CSRT and a diet program. The diet group received only a diet program, and the control group received none of these treatments. The evaluations (see measures section for details) were performed at three time points (i.e., before the beginning of the intervention [pretest], after the intervention [posttest], and a 4‐week follow‐up after the posttest). The same evaluation sessions were performed for diet and control groups as well in a similar time interval between the sessions. Because several participants withdrew their participation in different stages of the work, 56 participants finished all the steps of the work and were kept in our data analyses (see Table 1). The implementation steps of the main phase are presented in Figure 1.

**TABLE 1:** Participant characteristics per training condition

		Cognitive self‐regulation (*n* = 18, 18 females)	Diet (*n* = 17, 15 females)	Control (*n* = 21, 21 females)	Range
Age (mean [STD])		14.25 (0.78)	14 (1.84)	14.19 (0.89)	12–16
School grade	Seventh	5	7	8	
	Eighth	9	5	8	
	Ninth	4	5	5	
Anxiety (mean [STD])		5.25 (4.66)	5.19 (4.36)	4.7 (3.62)	0–9
Depression (mean [STD])		5.95 (4.33)	5 (3.78)	5.61 (4.05)	0–13
Stress (mean [STD])		7.65 (3.92)	7.42 (5.09)	7.11 (4.1)	0–18
Eating disorder (mean [STD])		11.6 (6.18)	11.42 (5.53)	9.69 (7.13)	5–19

*Note*: Means and standard deviations for baseline characteristics are provided per condition.

The study protocol was approved by the local Ethics Committee in Biomedical Research of Kharazmi University, Tehran, Iran, and the participants and their parents provided written informed consent. Written consent to participate in the study was obtained from participants and their parents.

### 2.3 Measures

#### 2.3.1 Depression Anxiety Stress Scale (DASS)

The Depression Anxiety Stress Scale (DASS) (Lovibond & Lovibond, 1995) is a 42‐item questionnaire, designed to maximize the identification of the distinct features of depression, anxiety, and stress. In this study, we have administered a validated Persian version of DASS. Cronbach's alpha was reported for depression, anxiety, and stress, respectively, as 0.93, 0.88, and 0.82 (Sahebi et al. 2005). The subject should mark the frequency of each group of symptoms that she/he has experienced during the last week. The scoring is based on the Likert scale and ranges between 0 and 3 (3 = “Almost Always,” 0 = “Never”). This test was only used as a screening test for subject inclusion.

#### 2.3.2 Eating Attitudes Test (EAT‐26)

The Eating Attitudes Test (EAT‐26) is a widely used standardized measure of symptoms that concerns the characteristics of eating disorders (Garner et al. 1982). The EAT‐26 questions are scored according to the Likert scale (3 = “Always,” 0 = “Never”). The scoring of the 26th question is the opposite. Scores higher than 20 indicate a need for further investigation by a qualified professional. Cronbach's α has been reported .94 in clinical and nonclinical groups (Garner et al., 1982). The validated Persian version of EAT‐26 is administered in the present study (Babai et al. 2008). This test was only used as a screening test for subject inclusion.

#### 2.3.3 Dutch Eating Behavior Questionnaire (DEBQ)

The DEBQ (Van Strien et al., 1968; Van Strien et al., 2005) is a 33‐item self‐report measure of emotional eating (Cronbach's α = .96), external eating (Cronbach's α = .77), and restrained eating (Cronbach's α = .90). All items are scored on a 5‐point Likert scale (1 = Never’’; 5 = “Very often”). This scale was administered in the pretest, posttest, and 4‐week follow‐up as an outcome measure.

#### 2.3.4 Body mass index

**FIGURE 2:**
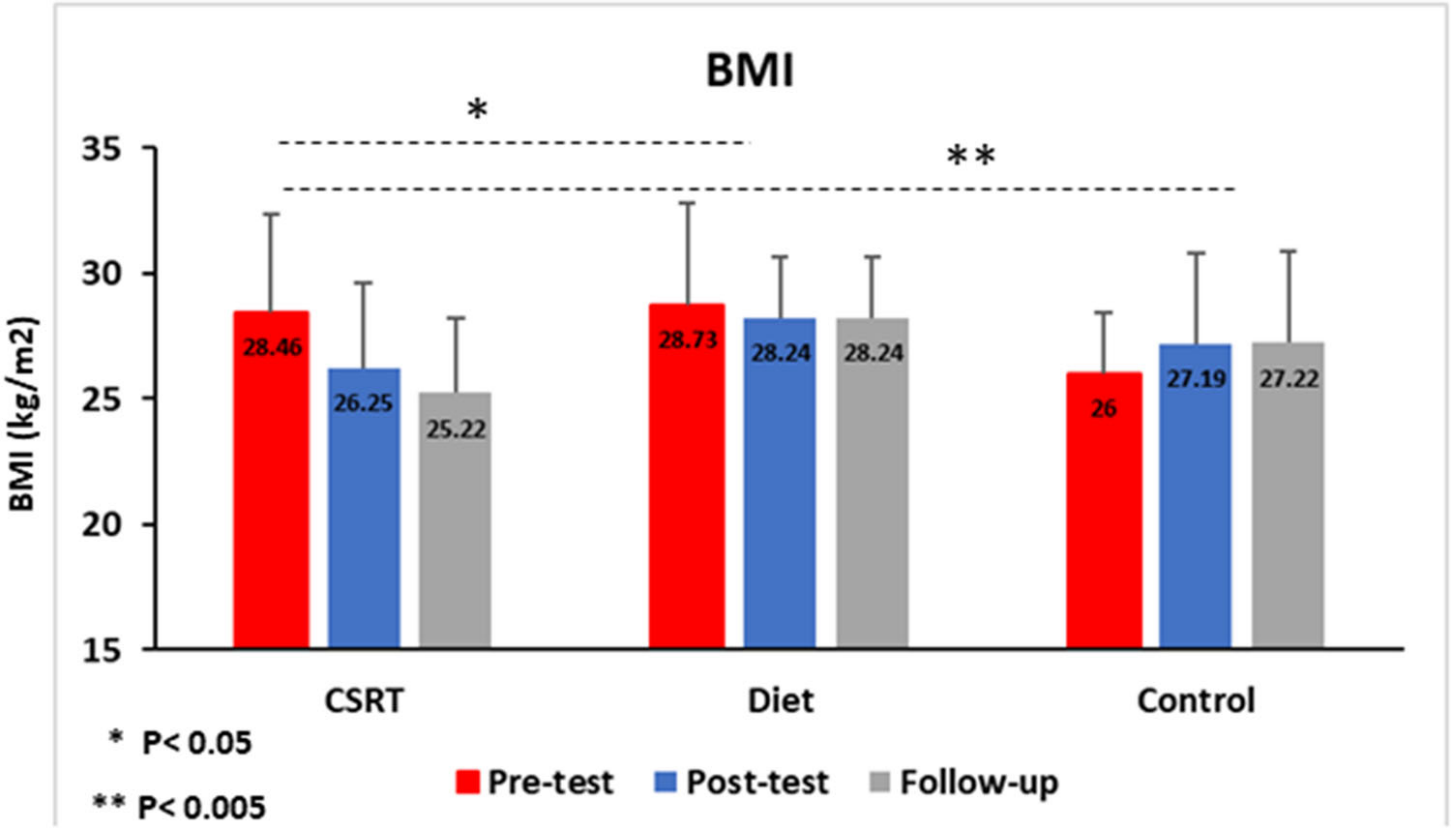
Data represent mean. Changes of BMI in different condition across time (pretest, posttest, and follow‐up). *p < .05; **p < .01. BMI, body mass index; CSRT, cognitive self‐regulation training

The Body Mass Index (BMI) (weight [kg]/height [m^2^]) was determined for each child pretest, posttest, and at 4‐week follow‐up. In order to make BMI comparisons between children of different ages, this study uses the adjusted BMI according to the criteria of the Centers for Disease Control and Prevention (CDC), and participants with a BMI ≥85th percentile for age and gender were included in the study (Kuczmarski et al., 2000). BMI was measured in the pretest, posttest, and 4‐week follow‐up as an outcome measure (see in Figure 2).

### 2.4 Intervention

#### 2.4.1 Cognitive self‐regulation training (CSRT)

This intervention was designed in our previous work (Rezaei, 2021), based on McCloskey and colleagues’ suggestions on the concept of executive skills and their role in self‐control and self‐regulation. According to McCloskey, “*The executive skills drive the perceptions, feelings, thoughts, and actions that are deemed necessary by the executive functions*.” (McCloskey et al., 2014). Three main components of executive functions have been the focus of the training program (inhibitory control, working memory, and cognitive flexibility). In addition, other components of executive functions such as self‐monitoring, initiation, maintenance, problem‐solving, metacognition, prioritization, and emotion regulation are also considered in this model. In this model, we taught participants how to use their executive skills to improve self‐control in eating behavior.

Participants in the CSRT group took part in a cognitive self‐regulation training program. The intervention was delivered by a cognitive psychology researcher and a nutritionist. The CSRT, which is administered in this study, consists of 10 steps (see Supplementary material 1 for a detailed program of CSRT). All steps follow a general framework; each step has a topic, according to which the participant is asked to perform a task. Each step is performed in one session. In this program, the participants are provided with information on cognitive functions and their role in weight management. In each topic, examples are provided to the participants so that they understand the concepts. The therapist, who is a licensed psychologist, must be sure that the participants are familiar with the brain and its functions. The session instructions are written in a way that is understandable to children and adolescents over the age of 10. In addition, after each session, there are homework tasks, some of which require the help of a parent or another person.

In this study, due to the Coronavirus (COVID‐19) pandemic and lockdowns, children's educational sessions were held online in two 0.5‐h sessions per week; 10 steps were performed in 10 weeks, making a total of 20 sessions for each participant of the CSRT group. In addition to training the child, there are four parent‐education sessions in this program; parent meetings were held in the first, third, sixth, and ninth week of the CSRT training. The parent education sessions included the role of parents in improving eating behavior patterns, creating, and maintaining the motivation to lose weight in their children, accompanying children to increase physical activities, familiarizing themselves with the weight loss procedure and not having unrealistic expectations and collaborating to follow the steps of the CSRT program. For the diet, both in CSRT and diet groups, personalized hypocaloric moderate‐carbohydrate diet plans were given to participants by a nutritionist. These diet plans have been used in our previous works (Shemirani et al., 2021).

### 2.5 Statistical analyses

Scores of eating behavior (including external, emotional, and restrained eating sub scores) and BMI were implemented in a mixed within‐between 2 × 3 repeated measures ANOVA with a between factor Group (CSRT, diet, and control), and within factor Time (pretest, posttest, follow‐up). We also tested for the interaction terms of Group × Time for each score.

Post hoc analyses with the Tukey's and Bonferroni tests have also been used to see the origin of the observed effects. We have removed all the data of the participants who withdraw their participation or did not follow the intervention plan at any stage of the work. All the statistical analyses were performed using the SPSS 26 software (Release notes—IBM® SPSS® Statistics 26.0).

It is important to note that we applied Levene's test for equality of variances, which resulted in *p* > .05 (i.e., the data have homogenous variances). Regarding the sphericity assumption, Mauchly's test resulted in a *p* < .05. We, therefore, used the lower‐bound and Greenhouse‐Geisser corrections to have a more conservative approach considering the assumption of sphericity is not met, and to reduce the potential Type 1 error rate in our results.

## 3 RESULTS

### 3.1 Demographic data

The demographic data of the participants are presented in Table 1. The three groups were matched for age, gender, and school grade. See Table 1 for details on demographic data.

### 3.2 BMI results

Repeated measures ANOVA showed main effects of Time (*F*(1, 53) = 5.18, *p* = .027, η^2^ = 0.09), Group (*F*(2, 53) = 6.07, *p* = .004, η^2^ = 0.18), and an interaction between Time and Group (*F*(2, 53) = 4.86, *p* = .012, η^2^ = 0.15).

Following the main effect of Time, a Bonferroni post hoc test has revealed significant differences between pretest and posttest (*p* = .009), and pretest and follow‐up (*p* = .042). However, there was no significant difference in BMI between posttest and follow‐up times (*p* = 1). To evaluate the differences of BMI between groups, Tukey's test showed significant differences between CSRT and diet groups (*p* = .04), and CSRT and control groups (*p* = .004). However, no significant difference in BMI between diet and control groups was seen (*p* = .72). More details on the directions of the effects can be seen in Table 2.

**TABLE 2:** Means and standard deviations for the dependent measures at pretest, posttest and one‐month follow‐up, and changes in weight and BMI follow‐up/pretest

		Cognitive self‐regulation			Diet			Control		
		Pretest	Posttest	Follow‐up	Pretest	Posttest	Follow‐up	Pretest	Posttest	Follow‐up
Weight (kg)	Mean (SD)	72.13 (9.57)	67.85 (9.39)	66.33 (8.09)	73 (13.47)	72.11 (13.59)	72.05 (13.34)	69.30 (6.82)	69.93 (7.51)	70.23 (7.65)
Adjusted BMI (kg/m^2^)		28.46 (3.87)	26.25 (3.37)	25.22 (3.02)	28.73 (4.04)	28.24 (4.25)	28.24 (4.25)	26.87 (2.45)	27.19 (3.61)	27.22 (3.69)
Emotional eating		31.16 (11.23)	24.66 (12.18)	22.11 (11.23)	26.41 (13.67)	26.94 (13.46)	27 (13.61)	27.80 (12.33)	30.76 (11.30)	30.80 (11.35)
External eating		52.02 (23.54)	30.72 (15.59)	28.66 (13.40)	38.88 (17.66)	39.11 (17.66)	39.11 (17.66)	46.09 (21.59)	46 (19.73)	45.85 (10.47)
Restrained eating		33.16 (7.43)	35.16 (7.49)	36.77 (7.16)	30.76 (7.16)	30.11 (7.59)	30.11 (7.59)	31.47(7.24)	31.66 (7.55)	31.57 (7.74)
Weight loss follow‐up/pretest (%)		–5.8 (8.04)			–1 (1.3)			1 (1.34)		
BMI reduction follow‐up/pretest (%)		–3.24 (11.38)			–0.49 (1.70)			0.35 (1.30)		

### 3.3 Emotional eating results

**FIGURE 3:**
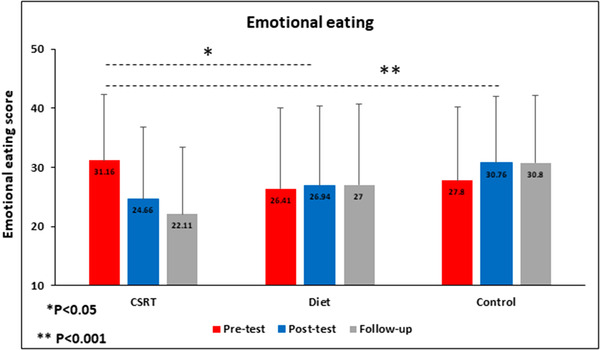
Data represent mean. Changes of emotional eating scores in different condition across time. *p < .05; **p < .01

Repeated measures ANOVA showed the main effect of Time (*F*(1.08,57.68) = 5.29, *p* = .023, η^2^ = 0.09), main effect of Group ([*F*(2, 53) = 25.29, *p* < .001, η^2^ = 0.48], and an interaction between Time and Group [*F*(2.17, 57.68) = 20.21, *p* < .001, η^2^ = 0.43]). Bonferroni post hoc tests showed no significant difference between pretest and posttest times (*p* = .317), while significant differences between pretest and follow‐up and between posttest and follow‐up times (*p* = .029 and *p* < .001, respectively). Post hoc analyses to elucidate differences in emotional eating scores between groups were done using Tukey's test. This test showed significant differences between CSRT and diet groups (*p* = .04), and CSRT and control groups (*p* < .001). However, no significant difference in emotional eating scores between diet and control groups was seen (*p* = .91). Figure 3 shows the results of emotional eating changes between groups over the three times. More details on the directions of the effects can be seen in Table 2.

### 3.4 External eating results

**FIGURE 4:**
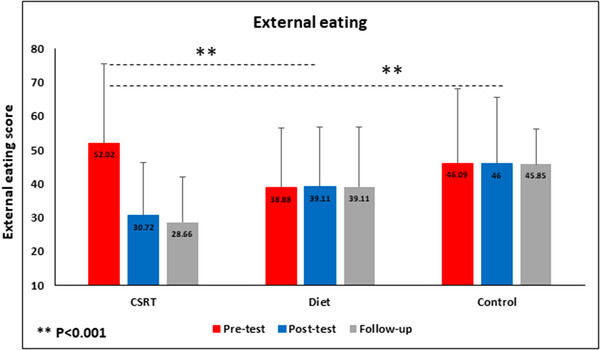
Data represent mean. Changes of external eating scores in different condition across time. *p < .05; **p < .01

Repeated measures ANOVA showed main effects of Time (*F*(1.03, 54.66) = 18.08, *p* < .001, η^2^ = 0.25), main effect of Group (*F*(2, 53) = 20.68, *p* < .001, η^2^ = 0.43), and an interaction between Time and Group (*F*(2.06, 54.66) = 18.00, *p* < .001, η^2^ = 0.40). Bonferroni post hoc tests showed significant differences in all three comparisons (i.e., between pretest and posttest time, pretest and follow‐up, and between posttest and follow‐up times (*p* = .001, *p* = .023, and *p* < .001, respectively). A series of Tukey's post hoc analyses on the differences in external eating scores between groups showed significant differences between CSRT and diet groups, and CSRT and control groups (both *p* < .001). However, no significant difference in external eating scores between diet and control groups was seen (*p* = .99). Figure 4 shows the results of external eating changes between groups over the three times.

### 3.5 Restrained eating results

**FIGURE 5:**
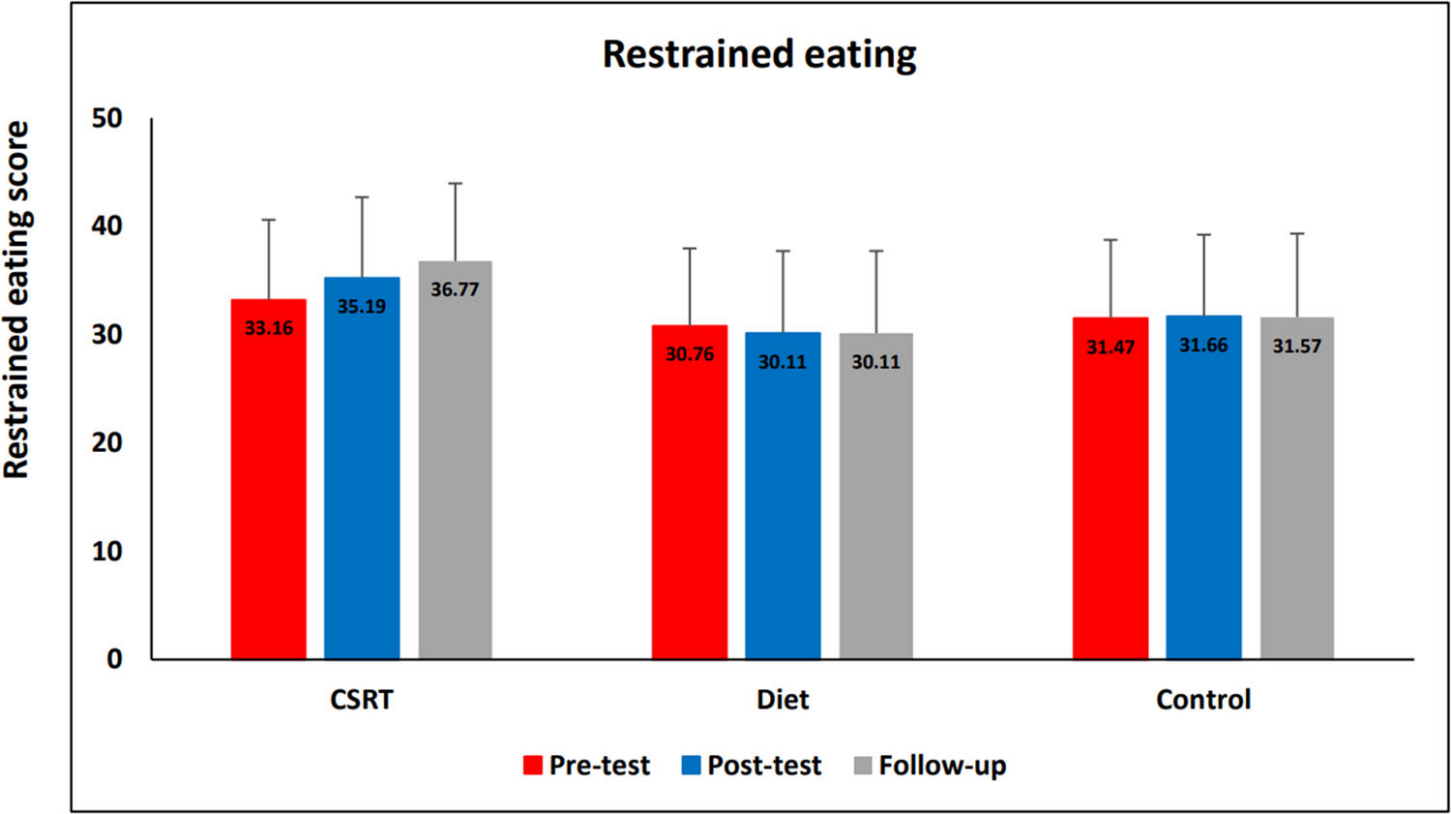
Data represent mean. Changes of restrained eating scores in different condition across time. *p < .05; **p < .01

Results of repeated measures ANOVA revealed neither main effect of Time (*F*(1.08, 57.47) = 1.39, *p* = .253, η^2^ = 0.02), nor interaction between Time and Group (*F*(2.16, 57.47) = 2.28, *p* = .10,, η^2^ = 0.79). However, it shows a trend for the effect of Group (*F*(2, 53) = 3.06, *p* = .06, η^2^ = 0.12). Figure 5 shows the results of restrained eating changes between groups over the three times.

## 4 DISCUSSION

This study aimed to evaluate an intervention model promoting self‐regulation of cognitive functions in order to modulate eating behaviors and weight control. We recruited adolescents with obesity and randomly assigned them to three groups: cognitive self‐regulation training (CSRT), diet, and control groups. Our results show that the participants who received CSRT had more BMI reduction than the other two groups (i.e., the diet and the control groups). We have observed significant decreases in emotional and external eating in the group of participants who received CSRT intervention compared to the other two groups.

An important therapeutic approach toward weight control is changing eating behaviors. This is because the change in eating habits and the type of relationship a person has with food modulates weight control (Davis et al., 2011; Verbeken et al., 2013; Yang et al., 2019). This approach can be done by improving executive functions, emotion regulation, and cognitive flexibility (Eichen et al., 2021; Favieri et al., 2019; Naets et al., 2018; Smith & Whittingham, 2017; Song et al., 2022). In this regard, a large number of studies have been performed, namely, family cognitive‐behavioral interventions (Tsiros et al., 2008), which improve working memory to reduce cravings (Verbeken et al., 2013), and aerobic exercise, which improves the executive functions in children and adolescents (Davis et al., 2011).

The CSRT of the present study was based on reducing reactivity to external cues in eating. This mainly includes modifying hunger training, strengthening inhibition behavior, managing eating behavior at parties, and changing eating habits. Successful reduction in response to the eating cues helps the person avoid excessive encounters with provocative eating situations. In fact, the situations where the person repeatedly needs to be restrained lead to an increase in cognitive burden and weakening executive function (Hofmann et al., 2012). This cognitive burden remains high as long as the person has not learned the ability of self‐control.

The rate of BMI reduction in the present work is higher compared to other psychological therapies performed in Iran for the treatment of childhood and adolescent obesity and overweight. For example, in a study based on cognitive‐behavioral therapy of the Cooper and Fairburn model (Cooper & Fairburn, 2001; Cooper et al., 2010) in the treatment of obesity in adolescent girls, a weight loss of on average 7% of the initial weight in 44 weeks was reported (Foladvand et al., 2012). This measure reached 8.4% of the initial weight in our study, though a shorter follow‐up. In another study based on the family cognitive behavior therapy in adolescents with obesity, the change in BMI is 1.36 (kg/m^2^) (Bayat, Rahimian Boogar, Talepasand, Yousefichaijan, 2014), whereas in the present study, this change reaches 3.24 (kg/m^2^). A similar study was conducted in Australia on children aged 12–18 years. The decrease of BMI in the intervention group is reported 1.6 (kg/m^2^) (Tsiros et al., 2008).

Reduction in emotional eating was another result of the CSRT in the present study. We have used several exercises in order to manage eating in emotional situations. For example, in the fifth step of the CSRT, the participants were given exercises to increase working memory capacity and to use this function to manage eating behavior. Increasing working memory capacity allows a person to record and manipulate information to achieve a specific goal (Baddeley, 1992; Baddeley & Logie, 2012; Cochrane, 2014). Regarding weight control and eating behaviors, improved working memory seems to allow a person to reappraise thoughts in tempting situations; as a result, one can substitute new behaviors.

Besides, teaching emotion regulation techniques also helps people manage their negative feelings and emotions by expressing them. In our CSRT experiment, people were taught to talk about the emotional states that cause them to overeat. Some studies suggest that emotional regulation is influenced by the maturation and enhancement of executive function components such as working memory (Chaplin & Aldao, 2013; Denham, 2006).

Promoting cognitive flexibility helps the individual identify fixed mental and behavioral frameworks, and learn to change them by changing attention control (Ochsner & Gross, 2005). Cognitive flexibility also influences the modification of the emotional regulation patterns (Johns et al., 2008). In this study, improving cognitive flexibility helped the participants to change eating behavioral habits and emotional eating.

It is noteworthy that when the changes of eating behavior are effective, there should be a decreased need in inhibition behavior. In fact, the individuals with obesity are relatively highly sensitive to their eating. They may therefore, show increased temptation for foods, which in turn leads to a high demand for inhibition (Bergman et al., 2003; Salehi Fadardi et al., 2013). This may explain the absence of changes in restrained eating behavior in our study; in this population the restrained eating behavior is already elevated, and we therefore, did not observe improvement in this scale.

It is worth noting that the present study has been performed during the Coronavirus (COVID‐19) pandemics and lockdown in Iran, which significantly changed the lifestyle and reduced physical activity, mainly in children and adolescents. Despite this, participants in the study performed the intervention successfully and were able to lose significant weight. A main limitation of the present study is lack of a long‐term follow‐up in order to verify the longevity of treatment effects. It is crucial to study both the short‐term and long‐term effects of cognitive training on weight control. It seems that performing further studies with longer follow‐up intervals may elucidate how long the effect of such training lasts. Another limitation of the present study is the relatively low number of participants. The small number of participants was the result of the lockdowns of COVID‐19 pandemics. In addition, several participants declined to continue their participation in different stages of the study. It is notable that individuals with lower BMI withdraw their participation from CSRT and diet groups, which in turn resulted in changes in the initial matching of the BMI of the three groups. Besides, it is important to note that we have not used an “intention to treat” approach. This approach minimizes risk of bias in data analyses (McCoy, 2017). In contrast, excluding participants who did not adhere to the intervention undermines the balance between the groups provided by randomization and increases risk of bias. This is because the individuals who adhere to the intervention are probably those who are already more motivated to lose weight or those who had a lower weight from the outset. Therefore, the reported results should be interpreted cautiously.

In conclusion, our study supports the view that changes in the executive function may lead to altered eating behavior and excessive weight gain in turn. The findings of this study suggest that CSRT may be an effective strategy for achieving improvements in body composition in adolescents and eating behaviors. The conceptualization of the origin of thoughts, feelings, and behavior using cognitive functions can help increase self‐regulation in changing inefficient eating behavior patterns.

## CONFLICT OF INTEREST

All authors declare no conflict of interest

### ETHICAL APPROVAL

The study protocol was approved by the local Ethics Committee in Biomedical Research of Kharazmi University, Tehran, Iran, and the participants and their parents provided written informed consent.

### PEER REVIEW

The peer review history for this article is available at https://publons.com/publon/10.1002/brb3.2772.

## Supporting information

Supplement Information

## Data Availability

The data that support the findings of this study are available from the corresponding author upon reasonable request.
