# Supplementary figures and images for: Is online self‐regulatory training effective in weight control? A pilot experiment on adolescence obesity during coronavirus‐19 lockdown
Source: Brain Behav. 2022 Oct 9;12(11):e2772. doi: 10.1002/brb3.2772 (PMC9660493; doi:10.1002/brb3.2772)

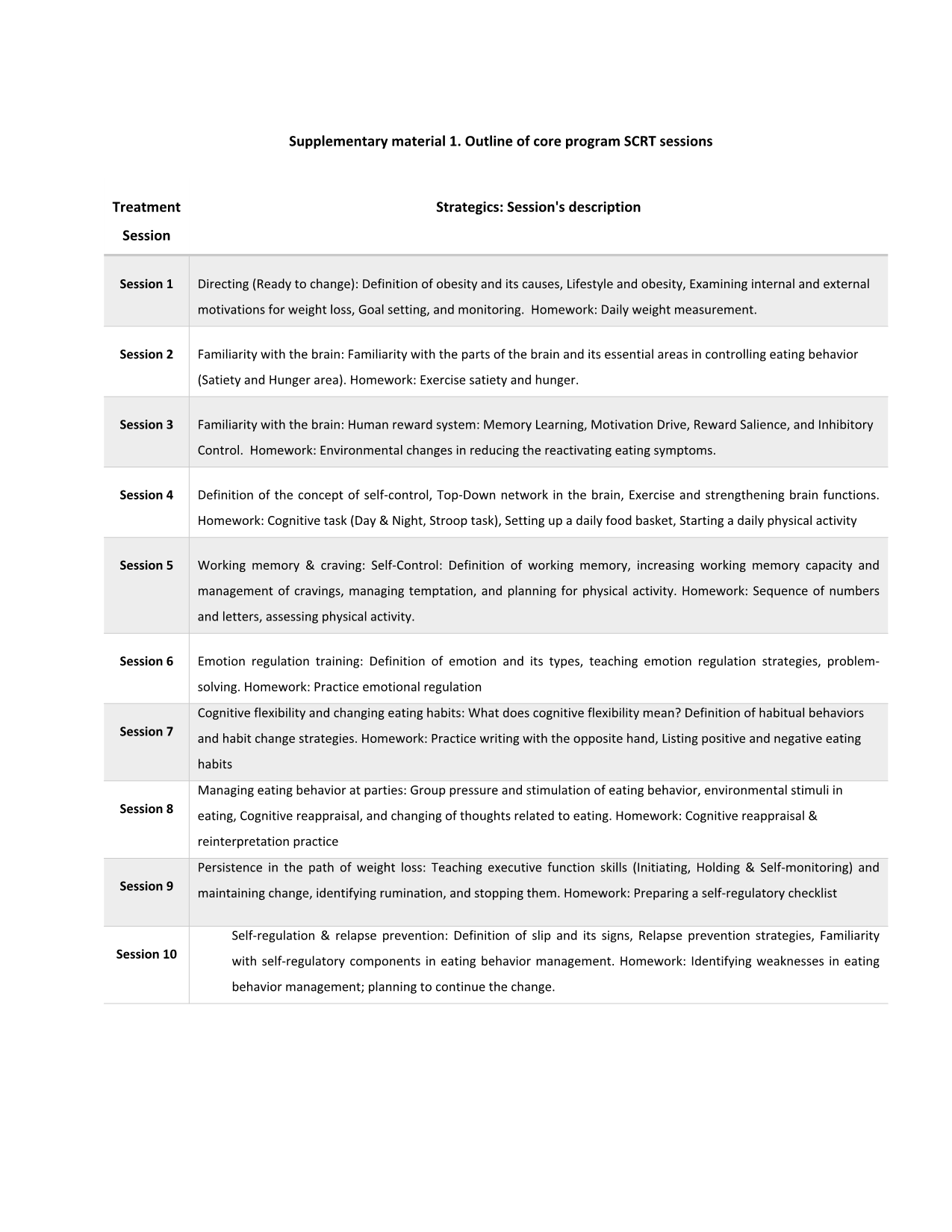

Supplement: Supplementary file 1 — Supplement Information [file BRB3-12-e2772-s001.tiff]
